# Prevalence and correlates of mental illness among inmates in North-western Ethiopia: A new look into the roles of rehabilitation service use

**DOI:** 10.3389/fpsyt.2022.983355

**Published:** 2022-11-18

**Authors:** Yassin Mohammed Yesuf, Amlaku Alemu Birhan, Addisu Gedlu Birara, Bewket Dereje Adimas, Abebe Bahiru Bezabh, Nega Gedefaw Agmase

**Affiliations:** ^1^Department of Psychology, College of Social Sciences and Humanities, University of Gondar, Gondar, Ethiopia; ^2^Department of Social Anthropology, College of Social Sciences and Humanities, University of Gondar, Gondar, Ethiopia; ^3^Department of Criminology and Criminal Justice, College of Social Sciences and Humanities, University of Gondar, Gondar, Ethiopia; ^4^Department of Law, School of Law, University of Gondar, Gondar, Ethiopia

**Keywords:** mental illness, inmates, prisons, rehabilitation services, North-western Ethiopia

## Abstract

Data on the magnitude of mental illnesses and associated factors among inmates in Ethiopia, in general and in the Amhara region in particular are scarce. The available studies either focused on specific type of mental illness or include inmates from a single correctional center and leave aside the role of rehabilitation service use in inmates’ mental illness. Therefore, the present study was conducted to look into the prevalence of mental illnesses and to examine the associated demographic, imprisonment related and rehabilitation service use related factors among inmates in Northwestern Ethiopia. The study employed cross-sectional, descriptive and explanatory research design where data was collected from 422 inmates from three randomly selected prisons. Inmates’ mental illness was assessed using the Self Reporting Questionnaire (SRQ-20). Frequency, percentage, bivariate and multiple logistic regressions were used to analyze the collected data. In the study it was revealed that 74.6% of the inmates in Northwestern Ethiopia have mental illnesses. Feeling unhappy, difficulty to play important role in life, headaches and bad sleep were experienced by majority of the respondents. Male inmates (AOR = 2.39, 95% CI = 1.07–5.37) and inmates who participate in the educational training services (AOR = 2.20, 95% CI = 1.36–3.55) were found to have higher chances of having mental illnesses. On the other hand, inmates who participate in life skill training programs (AOR = 0.45, 95% CI = 0.28–0.74) and inmates who participate in recreational and cultural activities (AOR = 0.26, 95% CI = 0.14–0.46) were found to have lower odds of developing mental illnesses. A high prevalence of mental illnesses among inmates was found in Northwestern Ethiopia and inmates’ participation in rehabilitation services were important correlates of their mental health. Thus, prison administrators and policy makers need to conduct large scale studies and develop tailored interventions that could reform the rehabilitation services provisions, including mental health service provisions.

## Introduction

Mental illness is now being recognized as a major public health problem throughout the world. Prevalence studies highlight the gravity of the problem and thereby challenge policy makers to take appropriate action. In Ethiopia mental illness comprised 11% of the total burden of disease and the disability associated with it is high (1).

Although no population group is immune to mental illnesses, some population groups are at higher risks of developing mental illnesses. The prison populations are among the high risk group of population for developing mental illnesses. In reality inmates were found to have higher levels of mental illnesses than the general population (2, 3).

The high prevalence of mental illnesses in the prison population could be attributed to pre-prison situations and/or to the prison environment. On the one hand, individuals with mental illnesses have higher chances of incarcerations for they are more likely to break the law (4). On the other hand, the prison environment characterized by overcrowding, violence, isolations, etc. could increase the probability of inmates developing mental illnesses (1, 4, 5).

Understanding the prevalence and associated factors of mental illnesses among prison inmates would help to provide targeted interventions and design appropriate health care services to the prison inmates (4, 6). Lack of such understanding could have dire consequences. Inmates with mental illnesses are at higher risk of suicide, self-harm violence, victimization, premature death and reoffending (3, 4). Therefore, it is imperative to examine the prevalence and associated factors of mental illnesses among prison inmates.

In practice, studies on the mental illness levels of inmates reported different prevalence rates ranging from 86% in Southwestern Uganda (2) to 29.2% in Zambia (7). With regard to contributors, a host of factors contributed to the high prevalence of mental illnesses among inmates. In resource poor countries like Ethiopia there are a host of factors that increased the risk of mental illness in prisons (1, 5).

While these are the facts on the ground, studies on mental illness prevalence and associated factors among inmates are scant in low and middle income countries (2, 7–10). Likewise, data on the magnitude of mental illnesses and associated factors among inmates in Ethiopia in general and in the Amhara region in particular are scarce (5, 6, 11). The available studies either focused on specific type of mental illness or include inmates from a single correctional center. For example two studies in Northwestern Ethiopia focused on psychological distress (6) and anxiety (12) while a study on mental illnesses of inmates in Northwestern Ethiopia collected data from Debremarkos correctional institute only (13). The focus on specific mental illnesses and specific facility will not give proper insights about the total picture of the mental illness situations of inmates to policy makers and prison administrators. In addition the studies examined the associated demographic and prison related factors which leaves aside the roles of important factors like participation in the available rehabilitation services.

Meanwhile studies in different corners of the globe have depicted rehabilitation service use related factors associated with mental illness of inmates. For instance, participation in life skill training programs (14, 15) and involvement in recreational and cultural activities (16–18) were associated with inmates’ mental illness.

Besides, the Ministry of Health considers the inadequate services in prisons, particularly the mental health service, as important factor that increased the risk of mental illnesses in prisons (1). Moreover, in a study in North-western Ethiopia it was depicted that low to no satisfaction with prison services significantly associated with inmates’ psychological distress level (6). These all implied that there is a need to examine the associations of service use with mental illness among inmates.

In the present study we argued that inmates’ participations in the rehabilitation services are associated with their mental illnesses. However, the association between prison rehabilitation service use and mental illnesses are least explored, at least to the knowledge of the present researchers. Hence, the present study was conducted to look into the prevalence of mental illnesses and to examine the associated demographic, imprisonment related and rehabilitation service use related factors among inmates in North-western Ethiopia. In doing so, the findings of the present study will help policy makers and prison administration bodies to plan targeted interventions, hire more mental health prison staff members and reform mental health service provisions.

## Materials and methods

### Research design

Based on its data collection timing, the present study employed cross sectional research design where data were collected at a time from all the respondents. In terms of its methods of analysis, the study employed both descriptive and explanatory research designs. It is descriptive in that it summarized and described the characteristics of respondents and their mental illness levels. It is explanatory because it tests the associations that exist between mental illness and potential predictor variables.

### Setting

Amhara regional state hosts 31 correction facilities from which 30 of them are under the administrative jurisdiction of the region and one (Shewarobit Rehabilitation and Correction center) is that of the federal government. Correctional centers in the region are divided into two levels: (a) Higher level (12 in number); and (b) medium and lower level (18 in number). Of the 30 prisons in Amhara National Regional State (excluding the center administered by the federal government), 10 of the prisons are found in the North-western part of the regional state. Simple random sampling technique was used to select three prisons: Gondar, Debretabor and Bahirdar prisons. Gondar and Bahirdar prisons are higher level correction centers while Debretabor correction center is included under lower and medium level centers. Based on the data collected from the three prisons there were 7,164 prison inmates. Specifically, there were 2,417 inmates in Gondar prison, 2,099 inmates in Debretabor prison and 2,648 inmates in Bahirdar prison.

### Sample size

For the purpose of determining the sample size of the study, single proportion formula was used because the total population was already known. Based on the computations using the formula, the minimum sample size was 384. Assuming 10% non-response rate, i.e., 38, the final sample size was 422.

Quota sampling is used to include proportional number of inmates from the three prisons. Simple random sampling was used to select participants from each correction center. Therefore, 142, 124, and 156 inmates from Gondar, Debretabor and Bahirdar prisons, respectively, were participated as questionnaire respondents in the present study. Inmates who were above 16 years and who were willing to participate in the study were included in the present study. On the other hand, inmates who were seriously ill and unable to communicate were excluded from participating in the study.

### Instrument

In the present study data was collected using a structured and pretested questionnaire. The questionnaire has four sections. The first section collects data about inmates’ demographic data (age, gender, educational status, religion, marital status and employment status before incarceration). The second section collects imprisonment related data that includes frequency of imprisonment, convict status, length of stay and types of crimes committed. The respondents were requested to report their number of imprisonment, convict status and the time they have stayed in prison. In terms of the crimes the inmates committed, the respondents list various kinds of crime. For ease of analysis, the types of crimes committed are categorized in to three: Crime against person, Crime against property and Crime against Society. Crimes like murder and rape are included under crimes against person. Crimes like automobile theft and robbery are categorized under crime against property. Crime against society includes crimes like human trafficking and corruption.

The third section includes lists of rehabilitation services (guidance and counseling services; life skill training program; educational training; vocational training; work experience/employment services; medical services; library services; recreational and cultural services; psychiatry services; social relation with family; and substance abuse treatment). Then, respondents were asked to indicate their participation in the listed services with “Yes, I participate” and “No, I don’t participate” options.

The fourth section of the questionnaire assess inmates’ mental illness level. Inmates’ mental illness was assessed using Self Report Questionnaire (SRQ-20) developed by WHO to be used in low income countries to assess mental illness symptoms (19). The SRQ was developed to assess 5 psychotic symptoms and 20 neurotic symptoms. The SRQ-20 which assessed 20 neurotic symptoms is used in the present study. Based on the user guide the inmates were presented with 20 statements and were asked to indicate if they have the typical symptom in the past month. They are also presented with Yes/No options and replying Yes (1) was considered as having the symptom while relying No (0) implying not having the symptom.

SRQ-20 has been used in prison settings in Africa. For example, it has been used in Zambia with a cut off score of > 8 (7). It has also been used among inmates in Ethiopia with different cut off scores. For instance, it was used in Debremarkos with a cutoff scores of ≥ 6 (13) while a study in Addis Ababa (10) and a study in Jimma correctional institute (9) used a cut off score of 8. In the present study a cut off score of 8 was used to categorize an inmate as having mental illness or not. In the present study SRQ-20 has been found to have high reliability with a Cronbach’s alpha score of 0.907.

### Data collection procedure

The questionnaire was translated to Amharic by a psychologist and language experts. It was then back translated by other psychologists and language experts who were not familiar with the purpose of the study. And minor differences in translations were resolved through a focus-group discussion.

Data collection process was carried out by six trained M.A. holders who are also the research team members. Two data collectors each visited the three prisons and collect data from the prisoners. Formal letters directed to the selected facilities were written from the college of social science and humanities at UoG requesting permission to collect data. While delivering the letters, the purpose of the research was vividly communicated to prison administrators.

Before data entry, collected data was examined and validated for completeness, and thereby incomplete data was eliminated to be replaced by other data.

### Data analysis

Both descriptive and inferential statistics were used in the present study. Frequencies and percentages were computed to describe respondents’ demographic, imprisonment related and rehabilitation service use related characteristics. Frequencies and percentages were also employed to describe prevalence of mental illnesses among inmates. To examine association between mental illness and the associated factors, bivariate and multiple logistic regression models were used. The statistical significance of mental illness and associated factors was determined using an adjusted odds ratio with a 95 percent confidence interval. All data analyses were carried out using SPSS version 23.

## Results

### Demographic descriptions of the respondents

As can be seen from Table 1 majority of the respondents are young aged between 18 and 40 years (74.2%), are males (92.2%), attend secondary education (39.3%), are Orthodox Christians (96%), are married (48.6%) and are employed (42.7%).

**TABLE 1:** Demographic characteristics of the respondents (*N* = 422).

Variables	Category	Frequency	Percentage
Age	18–40	333	78.9
	41–60	83	19.7
	>60	6	1.4
Gender	Male	389	92.2
	Female	33	7.8
Educational status	No education	47	11.1
	Primary education	147	34.8
	Secondary education	166	39.3
	Diploma	28	6.6
	Others	34	8.1
Religion	Orthodox	405	96.0
	Muslim	17	4.0
Marital status	Single	195	46.2
	Married	205	48.6
	Divorced	22	5.2
Employment status	Unemployed	63	14.9
	Employed	180	42.7
	Self-employed	179	42.4

### Imprisonment related characteristics of the respondents

Imprisonment related characteristics of the respondents are presented in Table 2. Majority of the respondents are imprisoned once (95.7%), have convicted status (85.5%), stay in the prison between 1 and 5 years (67.8%) and committed crimes against person (57.8%).

**TABLE 2:** Imprisonment characteristics of the respondents (*N* = 422).

Variables	Category	Frequency	Percentage
Frequency of imprisonment	First time	404	95.7
	Second time	8	1.9
	Third time	10	2.4
Convict status	Pre trail	23	5.5
	Accused	38	9.0
	Convicted	361	85.5
Length of stay	>1 Year	120	28.4
	1–5 years	286	67.8
	>6 Years	16	3.8
Types of crime	Crime against person	244	57.8
	Crime against property	123	29.1
	Crime against state	55	13.0

### Inmates’ participation at rehabilitation services

Inmates’ participation in the available rehabilitation services are presented in Table 3. Of the available services in the prisons, higher number of inmates participates in guidance and counseling service (66.1%); educational trainings (51.7%); vocational trainings (58.8%); work experience/employment services (54.3%); medical services (72.5%); and social relations with family (89.1%). Lower levels of participation was found at life skill training program (30.1%); library services (41.7%); recreational and cultural activities (14.9%); psychiatry services (13.3%); and substance abuse treatment (10.4%).

**TABLE 3:** Respondents’ rehabilitation service use status (*N* = 422).

Variables	Category	Frequency	Percentage
Guidance and counseling service	Use	279	66.1
	Don’t use	143	33.9
Life skill training program	Use	127	30.1
	Don’t use	295	69.9
Educational training	Use	218	51.7
	Don’t use	204	48.3
Vocational training	Use	246	58.3
	Don’t use	176	41.7
Work experience/employment services	Use	229	54.3
	Don’t use	193	45.7
Medical service	Use	306	72.5
	Don’t use	116	27.5
Library services	Use	176	41.7
	Don’t use	246	58.3
Recreational and cultural activities	Use	63	14.9
	Don’t use	359	85.1
Psychiatry services	Use	56	13.3
	Don’t use	366	86.7
Social relation with family	Use	376	89.1
	Don’t use	46	10.9
Substance abuse treatment	Use	44	10.4
	Don’t use	378	89.6

### Prevalence of mental illness among inmates

**FIGURE 1:**
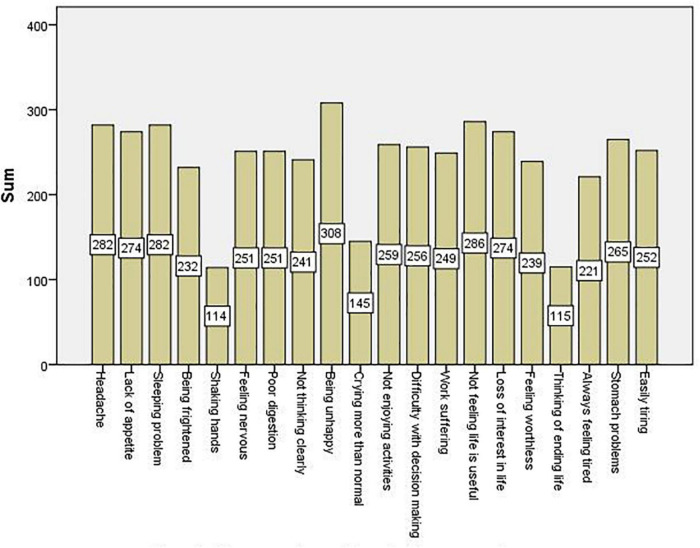
Frequency of reported mental illness symptoms.

Of all the participants of the study, 74.6% of them (374 in numbers) were found to have mental illnesses. Besides, feeling unhappy (308 inmates), unable to play useful part in life (286 inmates), head ache (282 inmates) and sleep badly (282 inmates) were symptoms reported by high number of inmates. On the other hand crying more than usual, suicidal thoughts and shaking hands were symptoms experienced by lower numbers of inmates, reported by 145, 115, and 114, respectively (see Figure 1).

### Factors associated with mental illness

Bivariate logistic regressions were computed to examine the association among inmates’ mental illness and the independent variables of the study (demographic variables, imprisonment related variables and rehabilitation service use related variables). In the bivariate analysis gender, length of stay, participation in life skill training program, use of educational training services and involvement in recreational and cultural activities were found to be significantly associated with inmates’ mental illness.

Following that multiple logistic regressions were computed to look into the individual contributions of each independent variable after adjusting for the effects of other potential predictor variables. In the multiple logistic regression analysis, all these variables, except length of stay in the prison, remained significant. In Table 4 variables which were significant in the bivariate analysis are included and data for other variables are not included (see Supplementary File 1). Specifically it was found that males were 2.39 times more likely to have mental illnesses (AOR = 2.39, 95% CI = 1.07–5.37) than female inmates. In terms of rehabilitation service use, inmates who participate in life skill training programs were found to have lower chances of developing mental illnesses (AOR = 0.45, 95% CI = 0.28–0.74) than inmates who don’t participate in life skill training programs. However, inmates who participate in the educational training services were found to have higher odds of having mental illnesses (AOR = 2.20, 95% CI = 1.36–3.55) than inmates who don’t participate in the educational training services. On the other hand, the odds of inmates developing mental illnesses were found to be lower for inmates who participate in recreational and cultural activities (AOR = 0.26, 95% CI = 0.14–0.46) than the inmates who don’t participate in these activities.

**TABLE 4:** Bivariate and multivariable logistic regression results of predictor variable.

Variable	Category	Mental illness		OR (CI)	*p*	AOR (CI)	*p*
		No	Yes				
Gender	Male	91	298	3.08 (1.50, 6.34)	0.002	2.39 (1.07, 5.37)	0.035
	Female	16	17	1			
Length of stay	>1 Year	38	82	1.66 (1.08, 2.54)	0.021	0.17 (0.02, 1.42)	0.102
	1–5 Years	68	218	0.14 (0.02, 1.13)	0.065	0.20 (0.02, 1.57)	0.124
	>6 years	1	15	1			
Life skill training program	Use	45	82	0.49 (0.31, 0.77)	0.002	0.45 (0.28, 0.74)	0.002
	Don’t use	62	233	1			
Educational training	Use	44	174	1.77 (1.13, 2.76)	0.012	2.20 (1.36, 3.55)	0.001
	Don’t use	63	141	1			
Recreational and cultural activities	Use	32	31	0.26 (0.15, 0.45)	0.000	26 (0.14, 0.46)	0.000
	Don’t use	75	284	**1**			

## Discussion

The present study explored the prevalence and correlates of mental illness among inmates in North-western Ethiopia. This study is one of its kinds in the sense that it considers rehabilitation service use as correlates of mental illness alongside demographic and imprisonment related factors.

In the present study it was revealed that about three quarter of the inmates in North-western Ethiopia have mental illnesses. A similar mental illness prevalence rate was reported in Iran where a prevalence rate of 73.9% was reported among prisoners (20).

Slightly lower prevalent rates of mental illness among inmates were reported in Ethiopia and other African countries. The prevalence of mental illness among prisoners in Debremarkos correctional institute was found to be 67.6% (13) slightly lower than the prevalence rate in the present study. The prevalence rate of mental illnesses among inmates in Addis Ababa was 58.4% (10). The prevalence rate of mental illnesses in Jimma correctional institute was 62.7% (9). A 63.2% prevalence rate of mental illness was reported among inmates from a study in Kenya (4). In a systematic review among studies in Africa, the pooled prevalence of mental illnesses among adults is 59% while it is 61% among the youth (8). Methodological differences and the characteristics of the respondents included in the studies are behind the differences in findings. For example, in the study in Addis Ababa half of the respondents (50.1%) are females while only 7.8% of the participants in our study are female inmates.

Higher prevalence rates of mental illnesses among prisoners than the prevalence rate in the present study was reported in other studies. For instance, in a study in South-western Uganda mental illness was observed in 86% of the inmates (2). Likewise, a study in India reported a mental illness prevalence rate of 83.5% (21). Psychological distress was identified among 83.4% of inmates among prisons in North-western Ethiopia (6). The differences in findings are attributed to the tools used to measure mental illnesses. The studies in Southern Uganda and India used Mini-International Neuropsychiatric Interview (M.I.N.I.) that measured the prevalence of psychotic disorders while the study in North-western Ethiopia employed K10 that examined psychological distress.

Significantly lower mental illness prevalence rates are also reported in other African countries. The prevalence of mental illness reported in Zambia is 29.2% (7). Similarly, a mental disorder prevalence rate of 34.8% was reported from a study in a correctional prison in Yaoundè, Cameron (22). The differences are attributed to differences in the settings the data is collected and the tools used to assess mental illness. For example the study in Zambia is conducted in maximum security prisons among remanded, sentenced, and condemned inmates. Besides, the study in Cameron used M.I.N.I. to assess mental illness while our study employed SRQ-20.

Alongside to reporting the prevalence of mental illness, the present study depicted the most and the list reported mental illness symptoms by inmates. Feeling unhappy, difficulty to play important role in life, headaches and bad sleep were experienced by majority of the respondents. These symptoms were reported as high in the study among prisoners in Jimma correctional institute, South-western Ethiopia (9). Hands shake, suicidal thoughts and crying more than usual were the symptoms least reported by inmates in the present study. These symptoms are also among the least reported symptoms in the study in South-western Ethiopia (9). These all could imply that the typical symptoms experienced by inmates are similar in different corners of the country.

Of all demographic variables considered, gender as an important correlate for inmates’ mental illness was found in the present study. Unlike the findings from other studies, the present study found that male inmates have higher odds of having mental illness than their female counter parts.

Contrary to our findings, the study at Debremarkos correctional institute found that female inmates have higher probability of having mental illness than male inmates (13). In a study in Bonga town correction center female inmates were found to feel worthless and nervous than male inmates (23). Similarly, in the study in Kenya female inmates were found to have higher chances of having mental illnesses than male inmates (4). The difference in the findings could be attributed to the high number of male inmates (92.2%) included in our study.

The present study highlights the important effects of rehabilitation service use on inmates’ mental illness. Of the 11 rehabilitation service use related variables considered in the study, three of them were found to be important correlates of inmates’ mental illness: life skill training program; educational training; and recreational and cultural activities.

In the present study, inmates who participated in the life skill training programs were found to have lesser chances of developing mental illness. Likewise, in a study in Iran the mental health of women inmates who participated in anger management trainings were improved after the training (14) indicating the importance of life skill trainings. Another study in Iran found that inmates who participated in life skill trainings were found to have higher scores in positive mental adjustment (measured by assertiveness and self-esteem) and lower score in negative adjustment (measured by anxiety, depression, stress and aggressiveness) (15).

Participations in recreational and cultural activities are found to have a buffering effect against mental illness among prisoners. In line with our finding, participation in recreational and cultural sport activities were reported as buffering against mental illnesses among inmates. For example, in a study in the USA inmates who participated in group activities were found to have lower chances of experiencing anxiety. On the contrary, the study depicted that being idle is associated with higher odds of anxiety and depression implying the importance of participation as a buffering against mental illnesses (17). In a study in Nigeria, inmates who participated in sport activities have better psychological and social wellbeing than inmates who don’t participate (16). Likewise, in a qualitative study in Northern Ireland it was depicted that participation in sport activities increases social interaction thereby improves inmates’ psychological wellbeing (18).

Surprisingly, in the present study, participation in educational training services was found to be associated with higher chances of having mental illness among inmates. In the literature, participation in prison educational programs was associated with lower recidivism, higher employment chances after release, reduced misconduct while in prison and strong return on investment (24). Findings of the present study associates prison education with increased mental illnesses. This could be potentially attributed to the many challenges associated to prison education in Africa, for example in South Africa (25) or the low quality and relevance of the educational service provisions reported among prisons in Amhara National Regional State (26) or other additional covariates that need further investigations.

## Conclusion, recommendations and limitations

A high prevalence of mental illnesses among inmates was found in Northwestern Ethiopia. Besides, inmates’ participation in rehabilitation services was important correlates of their mental health. The high rates of mental illness among the inmates calls up on an urgent intervention and prison mental health service reform to satisfy inmates mental health needs. Prison administrators, policy makers at regional and national levels, ministry of justice, and ministry of health as well as non-government organizations need to come together, discuss on potential immediate interventions and implement the interventions so as to combat the potential consequences of having such a huge number of inmates with mental illness in our prisons. Furthermore, the quality of the rehabilitation services rendered to the inmates need to be properly examined thereby appropriate measures need to be in place by concerned bodies.

Issues surrounding inmates’ mental health, including prevalence, types of illness, services that targeted mental illness, among other things are untapped research area for future researches. The role rehabilitation service use played against/toward inmates’ mental illness need detailed, potentially longitudinal, studies at a wider scale.

Finally, the facts that this study is conducted with only three prisons and its cross-sectional nature are the limitations of the study. Moreover, the numbers of female respondents in the present study are small in that it would be difficult to generalize the results to female inmates in the region. In addition, mental illness is not clinically diagnosed with trained professionals and there could be recall bias and/or over reporting of symptoms.

## Data availability statement

The original contributions presented in this study are included in the article/Supplementary material, further inquiries can be directed to the corresponding author.

## Ethics statement

The studies involving human participants were reviewed and approved by the College of Social Science and Humanities Research Review Committee. The patients/participants provided their written informed consent to participate in this study.

## Author contributions

YY, AAB, and BA conceived the study and developed the tool. AAB, BA, ABB, and NA collected the data. YY and AAB analyzed the data. YY, AAB, and AGB discussed the findings. YY, AAB, ABB, and NA developed the manuscript. All authors have read and approved the final manuscript.
