# Supplementary material for: Prevalence and correlates of mental illness among inmates in North-western Ethiopia: A new look into the roles of rehabilitation service use
Source: Front Psychiatry. 2022 Nov 18;13:983355. doi: 10.3389/fpsyt.2022.983355 (PMC9715590; doi:10.3389/fpsyt.2022.983355)
Supplement: Supplementary file 1 [file Data_Sheet_1.doc]

**Additional File 1**

Bivariate and Multivariate logistic regression results of demographic, imprisonment and service use related variables

| **Variable** | **Category** | **Mental illness** | | **OR (CI)** | **p** | **AOR (CI)** | **p** |
| --- | --- | --- | --- | --- | --- | --- | --- |
|  |  | **No** | **Yes** |  |  |  |  |
| Age | 18-40 | 86 | 247 | .00 (.00) | .999 |  |  |
|  | 41-60 | 21 | 62 | .00 (.00) | .999 |  |  |
|  | >60 | 0 | 6 | 1 |  |  |  |
| Gender | Male | 91 | 298 | 3.08 (1.50, 6.34) | .002 | 2.39 (1.07, 5.37) | .035 |
|  | Female | 16 | 17 | 1 |  |  |  |
| Educational Status | No education | 15 | 32 | .368 (.119, 1.139) | .083 |  |  |
|  | Primary | 30 | 117 | .672 (.240, 1.884) | .450 |  |  |
|  | Secondary | 50 | 116 | .400 (.146, 1.093) | .074 |  |  |
|  | Diploma | 7 | 21 | .517 (.144, 1.856) | .312 |  |  |
|  | Others | 5 | 29 | 1 |  |  |  |
| Religion | Orthodox | 104 | 301 | .62 (.17, 2.20) | .460 |  |  |
|  | Muslim | 3 | 14 | 1 |  |  |  |
| Marital Status | Single | 62 | 133 | .000 (.000) | .998 |  |  |
|  | Married | 45 | 160 | .000 (.000) | .998 |  |  |
|  | Divorced | 0 | 22 | 1 |  |  |  |
| Employment Status | Unemployed | 17 | 46 | 1.049 (.550, 1.999) | .885 |  |  |
|  | Employed | 40 | 140 | 1.36 (.84, 2.19) | .213 |  |  |
|  | Self-employed | 50 | 129 | 1 |  |  |  |
| Frequency of imprisonment | First time | 103 | 301 | .731 (.153, 3.496) | .694 |  |  |
|  | Second time | 2 | 6 | .750 (.081, 6.958) | .800 |  |  |
|  | Third time | 2 | 8 | 1 |  |  |  |
| **Convict status** | Pre trail | 2 | 21 | 3.64 (.84, 15.84) | .085 |  |  |
|  | Accused | 12 | 26 | .75 (.37, 1.55) | .440 |  |  |
|  | Convicted | 93 | 268 | 1 |  |  |  |
| Length of Stay | >1 year | 38 | 82 | 1.66 (1.08, 2.54) | .021 | .17 (.02, 1.42) | .102 |
|  | 1-5 years | 68 | 218 | .144 (.018, 1.129) | .065 | .20 (.02, 1.57) | .124 |
|  | >6 years | 1 | 15 | 1 |  |  |  |
| Types of Crime | Crime Against Person | 64 | 180 | 1.15 (.60, 2.21) | .665 |  |  |
|  | Crime against Property | 27 | 96 | 1.46 (.71, 3.00) | .305 |  |  |
|  | Crime Against State | 16 | 39 | 1 |  |  |  |
| Guidance and counseling Service | Use | 66 | 213 | 1.30 (.82, 2.05) | .263 |  |  |
|  | Don’t Use | 41 | 102 | 1 |  |  |  |
| Life skill training program | Use | 45 | 82 | .49 (.31, .77) | .002 | .45 (.28, .74) | .002 |
|  | Don’t Use | 62 | 233 | 1 |  |  |  |
| Educational training | Use | 44 | 174 | 1.77 (1.13, 2.76) | .012 | 2.20 (1.36, 3.55) | .001 |
|  | Don’t Use | 63 | 141 | 1 |  |  |  |
| Vocational training | Use | 69 | 177 | .71 (.45, 1.11) | .134 |  |  |
|  | Don’t Use | 38 | 138 | 1 |  |  |  |
| Work experience/employment services | Use | 54 | 175 | 1.23 (.79, 1.90) | .362 |  |  |
|  | Don’t Use | 53 | 140 | 1 |  |  |  |
| Medical service | Use | 72 | 234 | 1.40 (.87, 2.26) | .162 |  |  |
|  | Don’t Use | 35 | 81 | 1 |  |  |  |
| Library Services | Use | 53 | 123 | .65 (.42, 1.02) | .058 |  |  |
|  | Don’t Use | 54 | 192 | 1 |  |  |  |
| Recreational and cultural activities | Use | 32 | 31 | .26 (.15, .45) | .000 | .26 (.14, .46) | .000 |
|  | Don’t Use | 75 | 284 | **1** |  |  |  |
| Psychiatry Services | Use | 13 | 43 | 1.14 (.59, 2.22) | .693 |  |  |
|  | Don’t Use | 94 | 272 | 1 |  |  |  |
| Social relation with family | Use | 90 | 286 | 1.14 (.59, 2.22) | .693 |  |  |
|  | Don’t Use | 17 | 29 | 1 |  |  |  |
| Substance abuse treatment | Use | 9 | 35 | 1.86 (.98, 3.55) | .058 |  |  |
|  | Don’t Use | 98 | 280 | 1 |  |  |  |
